# The changing trends of HIV-1 prevalence and incidence from sentinel surveillance of five sub-populations in Yunnan, China, 2001–2010

**DOI:** 10.1186/s12889-015-1722-5

**Published:** 2015-04-12

**Authors:** Li Yang, Min Chen, Yanling Ma, Hongbing Luo, Chaojun Yang, Yingzhen Su, Huichao Chen, Yuhua Shi, Jingyuan Mei, Manhong Jia, Lin Lu

**Affiliations:** Institute for STD/AIDS Control and Prevention, Yunnan Center for Disease Control and Prevention, No 158, Dongsi Street, Xishan District, Kunming, 650022, Yunnan Province China

**Keywords:** Human Immunodeficiency Virus-1 (HIV-1), People who inject drugs (PWID), Male STD clinic attendees, Female Sex Workers (FSWs), Men who have sex with men (MSM), Pregnant Women, Prevalence, Incidence, Yunnan, China

## Abstract

**Background:**

Yunnan is one of the provinces hardest-hit by HIV in China. To understand HIV epidemic dynamics and evaluate prevention effectiveness, we studied the changing trends in HIV-1 prevalence and incidence among five sub-populations in Yunnan.

**Methods:**

Consecutive sentinel surveillances were conducted among people who inject drugs (PWID), male sexually transmitted diseases (STD) clinic attendees, and pregnant women for 2001–2010,female sex workers (FSWs) for 2007–2010, men who have sex with men (MSM) for 2008–2010. For the newly diagnosed HIV-seropositive samples, the recent infections were determined with BED-capture enzyme immunoassay (BED-CEIA), based on which HIV incidence was calculated for each sub-population using McDougal algorithm.

**Results:**

From 231,117 individuals, 6,107 HIV-positive samples were tested with BED-CEIA, among which 964 samples were identified as recent infections. In PWID, HIV prevalence for 2001–2010 was between 27.16% and 18.35%, while the estimated incidence rate significantly decreased from 11.68% in 2001 to 1.70% in 2010. Among male STD clinic attendees, both the HIV prevalence (from 3.62% in 2001 to 1.73% in 2010) and incidence (from 1.10% in 2001 to 0.40% in 2010) showed a significant decreasing trend. In FSWs, the HIV prevalence for 2007–2010 kept stable (between 2.46% and 1.95%), while the HIV incidence significantly decreased (from 0.71% in 2007 to 0.31% in 2010). In MSM, the HIV prevalence (between 11.78% and 9.42%) and incidence (between 6.01% and 8.38%) remained stable at a relatively high level for 2008–2010. In pregnant women, the HIV prevalence (between 0.44% and 0.30%) and incidence (between 0.15% and 0.08%) remained stable for 2001–2010.

**Conclusion:**

The HIV incidences in PWID, male STD clinic attendees and FSWs showed the decreasing trend, supporting a positive effect of prevention strategies for these sub-populations. MSM with the highest HIV incidence have become the sub-population most at risk. In most sub-populations, the HIV prevalence did not decline, suggesting the disease burden is still heavy. These findings are valuable for developing HIV prevention strategies in Yunnan.

## Background

Yunnan is located in Southwest China and situated along the drug trafficking routes channeling heroin into China from Southeast Asia’s opium-producing “Golden Triangle” region [1]. Since the first HIV epidemic in China was identified among people who inject drugs (PWID) in Yunnan in 1989, Yunnan has been one of the areas hardest hit by HIV in China [2]. By the end of 2010, the cumulative number of reported HIV infectors and AIDS patients (HIV/AIDS) in Yunnan was 83,925, accounting for 21.0% of the total national figure [3]. Furthermore, Yunnan serves as a primary entry point for the introduction of different HIV-1 genotypes into China [4-6]. Thus, Yunnan is accepted as an epicenter of the HIV-1 epidemic in China [2,7].

Usually, prevalence and incidence are used to describe the characteristic of HIV epidemic in a given population. Prevalence is the number of people living with HIV infection at a given time, including recent and long-term infections. Prevalence is most useful for measuring the burden of HIV in a population. However, incidence is the number of new HIV infections that occur during a given year. HIV incidence reflects the level of on-going HIV transmission, HIV infection trends, and the impact of HIV prevention efforts. Traditionally, HIV incidence estimation can be obtained through a prospective cohort study, in which participants are repeatedly tested. However, such surveillances are difficult to establish and costly to maintain. And cohort studies cannot avoid some of the biases inherent, such as the loss to follow-up and the change of participant behavior following risk-reduction counseling [8]. Alternatively, laboratory-based incidence assays have been developed since 1998 [9,10]. Among these methods, the BED-capture enzyme immunoassay (BED-CEIA) was the most commonly used incidence assay, which is based on measurement of the proportion of HIV-1-specific IgG to total IgG after seroconversion [11]. After the introduction of BED-CEIA into China in 2005, the correction factors were reset based on the characteristics of Chinese population [12]. The related studies showed that the HIV-1 incidence estimates yielded by BED-CEIA were similar to those obtained by cohort studies in China [12-14]. Yunnan was one of the provinces that earlier adopted BED-CEIA for HIV-1 incidence surveillance.

In China, the national comprehensive surveillance for HIV epidemic includes the national HIV sentinel surveillance system, HIV/AIDS case reporting system, and special epidemiologic surveys [15,16]. HIV sentinel surveillance is a series of cross-sectional surveys on risk behaviors and sero-testing in the representative areas and populations. The data from sentinel surveillance are used to analyze the trend of HIV prevalence and the risk factors for HIV infection in a giving region and population. The national sentinel surveillance sites have been established since 1995 [16]. With development, the national sentinel surveillance sites covers STD clinic attendees, female sexual workers (FSWs), PWID, long-distance truck drivers, pregnant women, paid blood donors, men who have sex with men (MSM), clients of female sex workers and tuberculosis (TB) patients [15,16]. To improve the coverage, some provincial sentinel surveillance sites were also established. These sentinel surveillance sites improved the ability to evaluate the HIV epidemic in China. Otherwise, the web-directed HIV/AIDS case reporting system started to run from March 2005. According to the regulation for HIV/AIDS cases reporting, all newly identified HIV/AIDS cases by confirmatory HIV testing are required to be reported into the system within 24 hours.

However, the present surveillance can not directly provide the HIV incidence, which is vital to understand HIV transmission dynamics and evaluate prevention effectiveness. To improve the utilization of the data, the combination of epidemiological surveillance and laboratory assay may be a good choice. In this study, BED-CEIA was used to detect recent infection with the samples from sentinel surveillance for 2001–2010 in Yunnan, including PWID, male STD clinic attendees, FSWs, MSM and pregnant women. This study explored the HIV prevalence and incidence trends from 2001 to 2010 in Yunnan, and is important to direct the future prevention and control efforts.

## Methods

### Sample collection and serological testing

By 2010, 90 sentinel sites were set up for regular surveillance among PWID (24 Sites; sample size: 400 per site), male STD clinic attendees (15 Sites; sample size: 400 per site), pregnant women (15 Sites; sample size: 400 per site), FSWs (32 Sites; sample size: 400 per site), and MSM (4 Sites; sample size: 200 per site) in Yunnan Province. Consecutive sentinel surveillances were conducted each year among PWID, male STD clinic attendees and pregnant women between 2001 and 2010, among FSWs between 2007 and 2010, among MSM between 2008 and 2010. Each participant received an anonymous interview to collect information on demographic details and HIV transmission related risk behaviors and provided 2–3 ml of whole blood. Plasma was separated from whole blood for HIV testing. Samples were screened for HIV-1 antibody with an enzyme immunoassay or rapid test, and then HIV-1 infection status was confirmed with a Western blot assay (HIV BLOT 2.2, MP Diagnostics, Singapore). All HIV tests were informed and voluntary. Written consents were obtained from all participants. The study was approved by Biomedical Ethics Review Committee of Yunnan Province.

### BED capture enzyme immunoassay

Excluding previously reported HIV-seropositive samples, the newly diagnosed HIV-seropositive samples were subjected to BED-CEIA testing. The BED-CEIA was performed according to the manufacturer’s instructions (Calypte HIV-1 BED incidence EIA, Calypte Biomedical Corporation, Portland, OR). Appropriate calibrator and negative control, low positive control and high positive control were run in triplicate for every plate. Test specimens were initially run singly. The optical density (OD) values of test specimens were normalized by a ratio using a calibrator (specimen OD/calibrator OD) to minimize inter-run variations. If the normalized OD (ODn) was >1.2, the specimen was classified as being from a long-term seroconverter. Specimens with ODn <1.2 were tested again in triplicate to confirm the values. In confirmatory testing, specimens with ODn values <0.8 were considered to have undergone recent seroconversion.

### Calculation of incidence

HIV incidence rate was calculated using McDougal formula [17]:

$$ I=\frac{F\times \left(365/w\right)\times R}{N+F\times \left(365/w\right)\times R/2}\times 100\% $$

Where *I* is the annual HIV-1 incidence; *F* is the correction factor; *w* is the window period (the mean length of time individuals remain classified as “recently infected” by BED-CEIA; in China, *w* = 168 days) [12]; *R* is the number of recent HIV infections detected by BED-CEIA; N is the total number of HIV-seronegative subjects.

The correct factor was calculated using the following formula [17]:

$$ F=\frac{\left(R/P\right)+\gamma -1}{\left(R/P\right)\times \left(a-\beta +2\gamma -1\right)}\times 100\% $$

Where *R* is the number of recent HIV infections detected by BED-CEIA; *P* is the total number of HIV-seropositive subjects that should be detected with BED-CEIA; α is the sensitivity of BED-CEIA for recent infection (<1 week); β is the specificity of BED-CEIA for the sample whose infected time between 1 week and 2 weeks; γ is the specificity of BED-CEIA for the sample whose infected time beyond 2 weeks. In China, α = 0.8098, β = 0.7571 and γ = 0.9315 [12].

When only a part of samples are detected by BED-CEIA among all the samples that should be detected, the number of recent HIV infections is adjusted using the following formula: R = R′ × (P′/P). Where R′ is the number of recent HIV infections actually determined by BED-CEIA, P′ is the number of HIV-seropositive samples actually detected with BED-CEIA, p is the number of HIV-seropositive subjects that should be detected with BED-CEIA.

The 95% confidence interval (CI) for the incidence estimate is given by the following formula:

$$ 95\%\mathrm{C}\mathrm{I}=\frac{\pm 1.96I}{\sqrt{R}} $$

According to the Operations Manual for HIV-1 Recent Infections Surveillance issued by Chinese Center for Disease Control and Prevention, the estimated incidence is unreliable if the number of recent infections identified by BED-CEIA is less than 10. For pregnant women, the number of recent infections for each year was less than 10, a solution was to combine the data of every two years to calculate the incidence. For the other four sub-populations, the incidences for each year were calculated.

### Statistical analysis

Statistical analyses were conducted using the SPSS 19.0 statistical analysis software package (SPSS Inc. Chicago, IL). Categorical variables were compared using χ^2^ tests. Trend tests were performed using χ^2^ tests with linear-by-linear association. All tests were two-tailed and a *p*-value <0.05 was considered statistically significant.

## Results

A total of 231,117 subjects were tested for HIV in sentinel surveillance program between 2001 and 2010, including 25,654 PWID, 48,125 male STD clinic attendees, 110,126 pregnant women, 45,604 FSWs (2007–2010) and 1,611 MSM (2008–2010). Through serum screening, HIV-1 antibodies were identified in 8,432 samples, in which 1,991 subjects had been reported as HIV-1 infection before participating in the sentinel surveillance program. All the newly reported HIV-1 infected subjects were confirmed with western blot assay. Except 334 samples whose volumes were not enough for BED-CEIA, a total of 6,107 samples (94.8%) were tested with BED-CEIA, among which 964 samples were identified as HIV-1 recent infection. Based on these, the prevalence and incidence estimates were obtained for each of the five sub-populations (Table 1).

**Table 1:** **HIV-1 prevalence and incidence from sentinel surveillance**

**Population**	**Year**	**The number of subjects**	**The number of HIV antibody-positive subjects**	**Prevalence rate (%,95%CI)**	**The number of previously reported cases**	**The number of samples tested with BED-CEIA**	**The number of recently infected individuals**	**Estimated HIV incidence rate (%,95%CI)**
PWID	2001	1344	365	27.16 (24.37 ~ 29.94)	0	345	72	11.68 (9.06 ~ 14.30)
	2002	1651	379	22.96 (20.64 ~ 25.27)	0	378	100	12.96 (10.42 ~ 15.50)
	2003	2212	475	21.47 (19.54 ~ 23.40)	9	464	52	2.74 (1.99 ~ 3.48)
	2004	2331	531	22.78 (20.84 ~ 24.72)	12	517	76	5.23 (4.06 ~ 6.40)
	2005	3008	561	18.65 (17.11 ~ 20.19)	33	503	55	2.07 (1.54 ~ 2.60)
	2006	2338	491	21.00 (19.14 ~ 22.86)	171	284	32	1.80 (1.21 ~ 2.39)
	2007	2416	571	23.63 (21.70 ~ 25.57)	223	331	39	2.18 (1.52 ~ 2.85)
	2008	2691	563	20.92 (19.19 ~ 22.65)	162	338	38	1.94 (1.38 ~ 2.51)
	2009	4626	1314	28.40 (26.87 ~ 29.94)	717	586	66	1.87 (1.42 ~ 2.32)
	2010	3037	633	20.84 (19.22 ~ 22.47)	318	307	38	1.70 (1.17 ~ 2.24)
Male STD clinic attendees	2001	3922	142	3.62 (3.03 ~ 4.22)	0	140	27	1.10 (0.69 ~ 1.52)
	2002	5222	122	2.34 (1.92 ~ 2.75)	0	120	27	0.88 (0.55 ~ 1.22)
	2003	5419	135	2.49 (2.07 ~ 2.91)	0	123	18	0.47 (0.26 ~ 0.68)
	2004	5627	113	2.01 (1.64 ~ 2.38)	3	101	18	0.52 (0.29 ~ 0.75)
	2005	5611	89	1.59 (1.26 ~ 1.92)	0	85	15	0.41 (0.21 ~ 0.62)
	2006	5636	88	1.56 (1.24 ~ 1.89)	0	60	10	0.37 (0.18 ~ 0.56)
	2007	4989	83	1.66 (1.31 ~ 2.02)	5	76	10	0.24 (0.09 ~ 0.38)
	2008	4956	67	1.35 (1.03 ~ 1.68)	5	55	8	0.23 (0.08 ~ 0.38)
	2009	3731	50	1.34 (0.97 ~ 1.71)	23	26	8	0.42 (0.13 ~ 0.70)
	2010	3012	52	1.73 (1.26 ~ 2.20)	19	32	7	0.40 (0.11 ~ 0.69)
FSWs	2007	11775	290	2.46 (2.18 ~ 2.75)	21	255	50	0.71 (0.52 ~ 0.90)
	2008	11604	226	1.95 (1.69 ~ 2.20)	12	202	32	0.40 (0.27 ~ 0.54)
	2009	12465	276	2.21 (1.95 ~ 2.48)	125	140	25	0.32 (0.20 ~ 0.44)
	2010	9760	217	2.22 (1.93 ~ 2.52)	105	112	20	0.31 (0.17 ~ 0.44)
MSM	2008	450	53	11.78 (8.61 ~ 14.95)	0	53	14	6.01 (2.86 ~ 9.16)
	2009	467	44	9.42 (6.64 ~ 12.21)	2	42	15	6.58 (3.25 ~ 9.90)
	2010	694	71	10.23 (7.85 ~ 12.61)	12	59	27	8.38 (5.22 ~ 11.54)
Pregnant women	2001-2002	20032	60	0.30 (0.22 ~ 0.38)	0	53	10	0.09 (0.04 ~ 0.14)
	2003-2004	23254	102	0.44 (0.35 ~ 0.52)	1	94	15	0.09 (0.05 ~ 0.14)
	2005-2006	23795	102	0.43 (0.35 ~ 0.51)	0	87	13	0.08 (0.04 ~ 0.12)
	2007-2008	26010	108	0.42 (0.34 ~ 0.49)	1	98	15	0.08 (0.04 ~ 0.12)
	2009-2010	17032	59	0.35 (0.26 ~ 0.43)	12	41	12	0.15 (0.07 ~ 0.22)

### PWID

The HIV prevalence and the estimated HIV incidence from 2001 to 2010 among PWID were shown in Table 1. The HIV prevalence did not show a decreasing trend (trend test, p = 0.051). However, the HIV incidence showed a significant decreasing trend for 2001–2010 (trend test, p < 0.01).

### Male STD clinic attendees

For male STD clinic attendees, the HIV prevalence and the estimated HIV incidence were shown in Table 1. Both the HIV prevalence and incidence among male STD clinic attendees showed a significant decreasing trend for 2001–2010 (trend test, p < 0.01).

### FSWs

In 2007, 14 additional FSWs sentinel surveillance sites were established, which improved monitoring HIV epidemic among this sub-population in Yunnan [7]. Thus, the FSWs surveillance data from 2007 to 2010 were used for analysis. From 2007 to 2010, the HIV prevalence and the estimated HIV incidence in FSWs were shown in Table 1. The HIV prevalence in FSWs kept stable (trend test, p = 0.431). However, the HIV incidence in FSWs showed a significant decreasing trend for 2001–2010 (trend test, p < 0.01).

### MSM

Province-wide MSM sentinel surveillance initiated in 2008. From 2008 to 2010, the HIV prevalence and the estimated HIV incidence in MSM were shown in Table 1. The HIV prevalence and incidence in MSM showed no significant change and remained stable at a relatively high level for 2008–2010 (trend test, p = 0.492 and p = 0.165).

### Pregnant women

For pregnant women, the surveillance data of every two years were merged to estimate the incidence from 2001 to 2010, the HIV prevalence and the estimated HIV incidence in pregnant women were shown in Table 1. The HIV prevalence and incidence in pregnant women remained stable for 2001–2010 (trend test, p = 0.552 and p = 0.190).

## Discussion

In this work, we first reported the province-wide application of BED-CEIA to estimate HIV-1 incidence among five sub-populations in China. To our knowledge, the study period of this work is the longest, and the sample size is the largest in China, which includes 10-year sentinel surveillance data and provides the trends of HIV-1 prevalence and incidence among five sub-populations for 2001–2010 in Yunnan, one of the provinces hardest-hit by HIV in China. The finding of this work is important to understand HIV-1 epidemic and evaluate the measures of HIV prevention and control in the first 10 years of this century in Yunnan.

In 1989, the first HIV epidemic in Yunnan was identified among PWID in Ruili County bordering Myanmar. Since then, PWID had become the prime force to drive the development of HIV epidemic in Yunnan [2,7]. Surveillance data showed that the average prevalence among PWID increased from 1992 to 1999 (2.7% in 1992, 15.0% in 1995 and 30.0% in 1999) [7]. The data from reported cases showed that the intravenous injection had been the premier transmission route until 2006 [2,7]. In this study, we found that the estimated HIV incidence rate among PWID remained around 12% from 2001 to 2002. In 2003, it significantly decreased. After 2005, it remained near 2%. The significant decrease of HIV incidence rate among PWID could result from the implement of the needle exchange programs (NEPs, introduced in 2002) and methadone maintenance treatment (MMT, introduced in 2004) [18-20]. Based on a mathematical transmission model, it was estimated that NEPs in Yunnan have averted approximately 16-20% (5,200-7,500 infections) of the expected HIV cases from 2002 to 2008 [21]. However, the prevalence among PWID remained stable at a high level during the study period, and was the highest among four high-risk groups. Moreover, HIV prevalence among PWID in Yunnan was two times of the national average level in this population for 2001–2010 [3]. The life cycle of PWID extended with the introduction of a nationally subsidized antiretroviral therapy program in 2004. Thus, the infected PWID would be repeatedly recruited in the sentinel surveillance. Meanwhile, more previously undetected infections were found with the expanding HIV testing in Yunnan. All of these could attenuate the contribution of the incidence decreasing.

A part of male STD clinic attendees were reported to visit FSWs [22]. As the important bridging populations, FSWs and male STD clinic attendees played an important role in driving sexual transmission and the transition of the epidemic from high-risk population to general population. Because of the limitation of surveillance data, the estimation of HIV epidemic in FSWs was performed only from 2007 to 2010 in this study. Nevertheless, the both HIV incidence rates among FSWs and male STD clinic attendees showed decreasing trend, which may related with the promotion of condom use and health education [23], which was introduced in 2002. According to the data of FSWs integrated intervention in Yunnan, HIV knowledge awareness rate increased from 82% in 2007 to 95% in 2010. More FSWs consistently used condom with clients in the last month (75% in 2007; and 86% in 2010) and in the last commercial sex (92% in 2007; and 94% in 2010). Male STD clinic attendees were the only population in which the HIV prevalence significantly decreased. Besides the decrease of incidence rate, there are other possible reasons. Because most STDs are curable, some STD clinic attendees infected with HIV would not return to STD clinic after their STDs were cured. This part of individuals would not be monitored in the following years, which means some long-term infectors were excluded when calculating the prevalence using the data from the sentinel sites of Male STD clinic attendees.

In recent years, a fast-spreading HIV epidemic among MSM brings a new challenge to China. The national HIV prevalence from MSM sentinel surveillance data showed a rising trend from 0.9% in 2003 to 6.3% in 2011 [24]. In this study, we found HIV prevalence from MSM sentinel surveillance in Yunnan was about 10% from 2008 to 2010, which suggested that Yunnan bears higher HIV burden in the MSM population. Strikingly, the HIV incidence in MSM was the highest among the five sub-populations and reached 8.38% in 2010. Although the HIV prevalence and incidence in MSM showed no significant change for 2008–2010, based on the HIV/AIDS case reporting system, the annual proportion of newly reported HIV cases attributed to homosexually transmitted infection in Yunnan increased (6.1% in 2008, 9.1% in 2009 and 12.0% in 2010), which may result from scaling up HIV testing among this population. Usually, MSM are difficult to access because of their underground behaviors and mobile lifestyles. From 2007, the intervention provided by peer educators from community based organizations was introduced, which promotes HIV testing, active care and antiretroviral therapy of HIV-positive MSM [25]. Presently, the epidemic in MSM is in the ascending period, more attention should be given to this sub-population.

HIV prevalence in pregnant women can be representative of that in the general population [26]. The data from pregnant women had been used to compare the regional variation of HIV epidemic [27,28]. Our study found that the HIV prevalence and incidence in pregnant women showed no significant change for 2001–2010, which suggested that HIV transmission between spouses and sexual partners was relatively stable.

According to the data of reported cases, the main transmission route changed from intravenous injection to sexual contact after 2006 [7]. According to the HIV/AIDS case reporting system, among the annual newly reported HIV cases, the proportion of the individuals infected by heterosexual contact continually increased from 30.6% in 2006 to 58.4% in 2010. However, the increase of the proportion of the sexual transmission did not convince the decreasing incidence observed in sentinel surveillance from the heterosexual transmission related populations, such as FSWs and male STD clinic attendees. The possible explanation may be that the HIV epidemic has transited from the high-risk populations to the general population, in which non-commercial sexual behaviors might be more prevalent than commercial sexual behaviors. Because the scale of the general population is larger than that of the high-risk populations, even if the incidence is low, the number of HIV-infected individuals will be larger. Furthermore, the more HIV-infected individuals previously undetected were found with increasing HIV testing(The total annual number for HIV testing increased from 95,755 in 2001 to 2,766,106 in 2010). Thus, the recent infection research for annual reported cases may provide more information to evaluate the epidemic in general population.

In fact, when using BED-CEIA, the misclassification of established infections as recently acquired infections may occur due to false-positive tests resulting from various reasons, such as antiretroviral therapy, low CD4^+^ T lymphocytes counting and HIV-1 genotypes [29]. To correct the potential disturbance, two strategies have been adopted. One is case-based adjustment, to exclude long-term infections by using additional information, such as the recorder of previous HIV testing and diagnosis and the number of CD4^+^ T lymphocytes. The other is formula-based adjustment using local correction factors for incidence estimates. In this study, although these two strategies have been considered, the misclassification may not be completely avoided. However, what we really concern is the changing trend of HIV incidence, which directly reflects HIV transmission dynamics and prevention effectiveness. With the consistent standard, the consecutive sentinel surveillances and BED-CEIA testing could provide the trend of HIV incidence.

## Conclusions

In conclusion, our study elucidated the trend of HIV prevalence and incidence for 2001–2010 among five sub-populations in Yunnan. HIV incidences among PWID, male STD clinic attendees and FSWs showed the declining trends, which suggested that the prevention and control efforts for these population should be continued. However, HIV incidence in MSM showed no significant change and remained stable at a relatively high level, which suggested that more resource should be input and the effective measures for HIV prevention should be developed for this population.
